# Vericiguat in patients with heart failure across the spectrum of left ventricular ejection fraction: a patient-level, pooled meta-analysis of VITALITY-HFpEF and VICTORIA

**DOI:** 10.3389/fendo.2024.1335531

**Published:** 2024-03-08

**Authors:** Chao Chen, Jin Lv, Changzhao Liu

**Affiliations:** Cardiovascular Disease Center, The Central Hospital of Enshi Tujia and Miao Autonomous Prefecture, Enshi Clinical College of Wuhan University, Enshi, Hubei, China

**Keywords:** Vericiguat, full range LVEF, heart failure, VITALITY-HFpEF, VICTORIA

## Abstract

Vericiguat, the newest soluble guanylate cyclase (sGC) drug, is potentially beneficial in treating heart failure (HF). However, most studies have only confirmed the significant impact of sGC in patients with reduced left ventricular ejection fraction (LVEF). Therefore, the main objective of this meta-analysis was to comparatively analyze the effects of Vericiguat in the entire LVEF range based on previous studies. According to PubMed, Web of Science, Cochrane, and Embase databases, randomized controlled studies in the full LVEF stage range were screened, and two extensive clinical studies on Vericiguat, namely VICTORIA (LVEF<45%) and VITALITY-HFpEF (LVEF≥45%) were identified for analysis and systematic evaluation. We separately assessed the rates of primary outcomes, cardiovascular death, and serious adverse events in both studies. The results of our research confirmed that although the criteria for the primary outcome were not the same in the two extensive studies, it was evident that there was no difference in the primary outcome between the experimental Vericiguat group and the placebo group in the VITALITY-HFpEF (LVEF≥45%) (P=0.45), whereas the primary outcome of VICTORIA (LVEF<45%) was significantly improved with the administration of Vericiguat showing a significant improvement (RR 0.93; 95% CI 0.87 to 1.00), but the effect of Vericiguat on cardiovascular mortality was not significant across the full range of LVEF (RR 0.97; 95% CI 0.86 to 1.09), and the incidence of total serious adverse events did not differ significantly between the two studies (RR 0.96; 95% CI 0.89 to 1.03). Surprisingly, partial subgroups analysis of serious adverse events found that vericiguat treatment reduced the incidence of all-cause death, Cardiac disorders, Hypotension, and Hypertension in patients with LVEF<45%, with a particular effect on the incidence of Cardiac disorders. Taken together, Vericiguat had a significant benefit in HF patients with LVEF<45%, especially in patients with LVEF<24%; it had a less pronounced effect in HF patients with LVEF ≥45%, but no adverse effects were observed.

## Introduction

Approximately 65 million adults worldwide have heart failure (HF), and the incidence and prevalence are expected to continue to increase in the coming decades (1, 2); HF continues to have one of the highest morbidity and mortality rates globally (3). Despite recent advances in the management of HF, the risk of death and hospitalization remains high in the long term (4). The search for effective therapeutic agents is critical. Left ventricular ejection fraction (LVEF) is used to indicate cardiac function. A healthy individual typically has a systolic relative to end-diastolic volume per beat ranging from 50% to 70%. With a reduced LVEF, the heart cannot pump enough blood to the body (5). Patients with HF were categorized into three groups based on their LVEF (1): HF with ≤40% reduction in LVEF (HFrEF) (2), HF with a mild reduction in LVEF of 41%-49% (HFmrEF) (3), HF with preserved LVEF ≥50% (HFpEF) (6). Because few patients in the HFmrEF range are not easy to study, studies in heart failure have generally focused on patients with HFrEF and HFpEF. In contrast, in the clinical studies included in this meta-analysis, VICTORIA enrolled patients with LVEF<45% and VITALITY-HFpEF enrolled patients with LVEF ≥45%, including both patients in HFmrEF range (7, 8), ESC Heart Failure found that HFrEF had a higher mortality rate than HFpEF after a 1-year follow-up (9). To provide better and more appropriate treatment to HF patients with different LVEF ranges, it is essential to clarify the therapeutic effects of a class of drugs on different LVEF stages.

Soluble guanylate cyclase (sGC) stimulators are a new class of drugs that have been recently studied for their emerging role in HF (10, 11). Cardiac characteristics of patients with HF include endothelial dysfunction, increased inflammation, and oxidative stress due to reduced activity of the nitric oxide (NO) sGC -cyclic guanosine monophosphate (cGMP) signaling pathway (12–14). Vericiguat is a novel drug that stimulates the cGMP pathway through direct and indirect stimulation of sGC (15). The beneficial effects of Vericiguat on cardiac remodeling and arrhythmia were confirmed in a mouse model of infarction (16). However, there are few studies on the beneficial impact of Vericiguat. It is effective in improving cardiac function in patients with reduced LVEF (17, 18). In our meta-analysis, Two extensive clinical studies, VICTORIA (LVEF <45%) and VITALITY-HFpEF (LVEF ≥45%), were selected by screening the literature; a total of 5036 patients were enrolled in the VICTORIA (LVEF <45%) study, of whom 2516 were in the experimental Vericiguat group and 2520 in the placebo group (LVEF ≤24%: 1472; LVEF 25-33%: 1871; LVEF 34-45%: 1693). The total number of patients in the VITALITY-HFpEF (LVEF ≥45%) study was 789, including 526 patients in the Vericiguat group and 263 patients in the placebo group (LVEF45-50%:168; LVEF50-60%:321; LVEF≥60%:299). The aim was to comparatively analyze the beneficial effects of Vericiguat in patients with HF in different LVEF ranges to identify target patients for whom Vericiguat exerts the most helpful treatment.

## Methods

This Meta-analysis utilized PubMed, Web of Science, Cochrane, and Embase databases to assess the role of Vericiguat in all patients within the LVEF range. And screening for selection of appropriate randomized controlled trials. The trials selected for this study were confirmed for availability by all authors. The primary outcomes assessed were primary outcomes or cardiovascular mortality outcomes or serious adverse events, among others. And by evaluating the continuous results of the data provided in the trial (including mean, standard deviation, number of events, and number of study participants). The statistical method used for this analysis most was fixed inverse variance. And we assessed the risk ratio for serious adverse events by the Cochran-Mantel-Haenszel test for the safety analysis to comparisons were made between the endpoint study values of the treatment groups receiving Vericiguat medication and placebo. Statistical significance was defined as a probability value of *P* ≤ 0.05. Statistical analysis was performed using RevMan 5.3 (Cochrane Collaboration, Oxford, United Kingdom).

### Literature search and eligibility criteria

**Figure 1:**
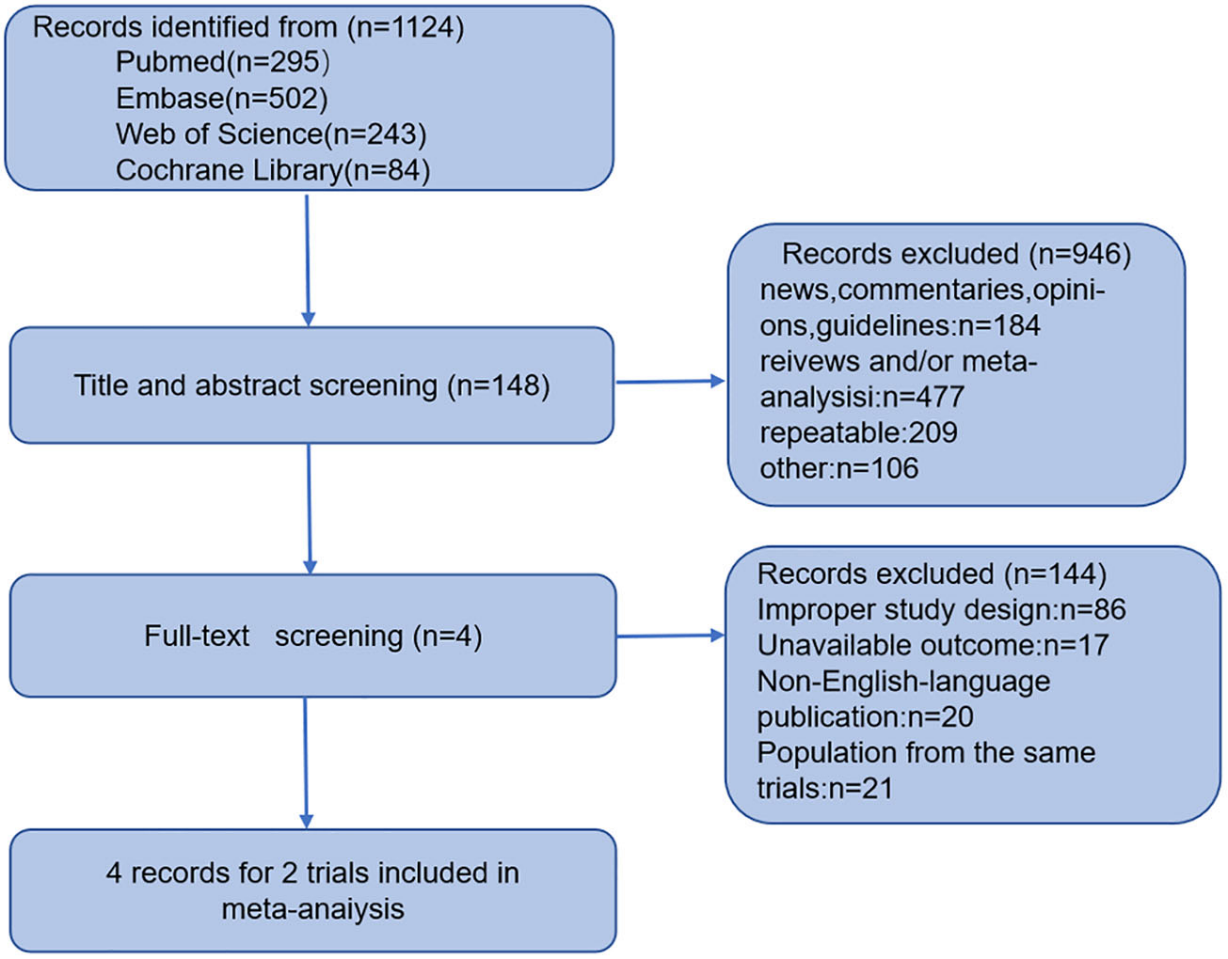
Flow chart of study selection.

The study screening process is shown in the flow chart in **Figure 1**. In PubMed, Web of Science, Cochrane, and Embase databases with the following search strategy: “LY3298176” OR “Vericiguat” AND “cardiovascular mortality” OR “heart failure” OR “cardiovascular disease” OR “cardiovascular death” OR “atrial fibrillation” OR “myocardial infarction (MI)” OR “coronary heart disease (CHD) events” OR “CVD events” OR “stroke.” From the 1124 articles initially identified, we discarded 946 by screening titles and abstracts. We further assessed 148 essays by reading through the full text. We used four studies related to two extensive clinical studies, VICTORIA (LVEF <45%) and VITALITY-HFpEF (LVEF≥45%), that were eligible for quantitative analysis.

## Results

### Baseline characteristics

Characteristics of VICTORIA and VITALITY-HFpEF Trials Studies as shown in **Table 1**, VICTORIA was a global clinical trial that enrolled a total of 5,050 patients and ended up with 4,872 actual participants, all with LVEF of 45% or less, and NYHA functional class II-IV. VITALITY-HFpEF included 789 patients from 21 countries, 735 final participants (LVEF≥45%), and NYHA functional class II-III. The two clinical studies included patients from the entire LVEF range. Both studies showed that patients with LVEF≥45% were older than those with LVEF<45%. Besides the high Systolic Blood pressure in patients with LVEF≥45% may be related to their older age. Body mass index (BMI) was approximately 3 kg m^-2^ higher in patients with LVEF≥45% compared with those with LVEF<45%, in terms of gender, which was most likely caused by the large proportion of women in patients with LVEF≥45% in **Table 2**. In addition, a history of hypertension and atrial fibrillation was more common, and a history of Coronary artery disease was less common in patients with LVEF≥45%, which may be related to the large proportion of smoking history in patients with LVEF<45%. In contrast, the history of Diabetes, Anemia, and Chronic obstructive pulmonary disease accounted for almost the same percentage of patients with LVEF in all stages.

**Table 1:** Characteristics of VICTORIA and VITALITY-HFpEF Trials Studies.

Study,Year (Reference)	Country	Median Follow up	Intervention	Patients n	Mean Baseline LVEF,%	Inclusion Criteria for Heart Failure
VICTORIA 2021 (19)	Global Study	Up to 6 mo	vericiguat Placebo	4872 (5050)	28.9±8.3 (<45)	LVEF<40% and NYNA functional class II-IV
VITALITY-HFpEF2022 (7)	21 countries	Up to 6 mo	vericiguat Placebo	735 (789)	56.3±8.0 (≥45)	LVEF≥45% and NYNA functional class II-III

**Table 2:** Characteristics of the patients at baseline.

LVEF (%)	≤24% (1472)	25-33% (1871)	34-45% (1693)	≥45% (789)		
				45-50% (168)	50-60% (321)	≥60% (299)
Placebo	751	921	848	59	102	101
Vericiguat	721	950	845	110	219	197
Age (years)	67.3 (12.2)			72.7 ( 9.4)		
Sex: No. (%)						
Female	284 (19.3)	444 (23.7)	477 (28.2)	385 (48.8)		
Male	1188 (80.7)	1427 (76.3)	1216 (71.8)	404 (51.2)		
Race, No. (%)						
White	859 (58.4)	1,201 (64.2)	1,169 (69.0)	674 (85.4)		
Black	125 (8.5)	77 (4.1)	46 (2.7)	21 (2.7)		
Asian	306 (20.8)	429 (22.9)	395 (23.3)	75 (9.5)		
other	182 (12.4)	163 (8.7)	83 (4.9)	19 (2.4)		
Region, No. (%)						
Americas	431 (29.3)	519 (27.7)	493 (29.1)	155 (19.6)		
Europe	489 (33.2)	636 (34.0)	564 (33.3)	562 (71.2)		
Asia Pacific	315 (21.4)	440 (23.5)	426 (25.2)	72 (9.1)		
Latin and South	237 (16.1)	276 (14.8)	210 (12.4)	0		
Body mass index, mean (SD)	26.7 (4.8)	27.2 (5.3)	27.5 (5.6)	30.7 (6.1)		
Blood pressure, mean (SD), mm Hg						
Systolic BP, mm Hg	115.1 (14.1)	118.7 (14.8)	124.4 (17.1)	129.4 (12.6)		
Diastolic BP, mm Hg	72.4 (11.1)	72.4 (11.1)	72.6 (11.1)	72.9 (10.5)		
Smoking history, No. (%)	926 (62.9)	1,084 (57.9)	955 (56.4)	342 (43.3)		
Clinical history No. (%)						
Hypertension	1,076 (73.1)	1,476 (78.9)	1,432 (84.6)	729 (92.4)		
Atrial fibrillation	590 (40.1)	823 (44.0)	849 (50.1)	485 (61.5)		
Coronary artery disease	990 (67.2)	1476 (78.9)	1322 (78)	362 (45.9)		
Diabetes	665 (45.2)	878 (46.9)	818 (48.3)	358 (45.4)		
Anemia	256 (17.4)	410 (21.9)	401 (23.7)	169 (21.4)		
Chronic obstructive pulmonary disease	226 (15.4)	322 (17.2)	316 (18.7)	154 (19.5)		
Heart rate, mean (SD), /min	73.7 (13.4)	72.4 (12.6)	71.1 (12.6)	70.4 (10.7)		
eGFR, mean (SD), mL/min/1.73 m2	62.3 (27.4)	58.3 (27.2)	56.7 (26.2)	59.5 (20.7)		

eGFR, estimated glomerular filtration rate; SD, standard deviation.

### Effect of vericiguat on outcomes according to ejection fraction

**Figure 2:**
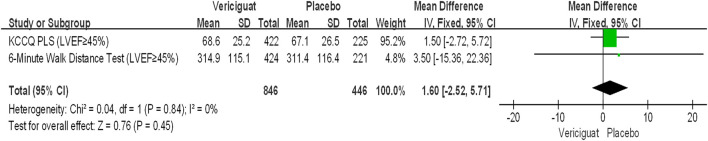
Primary outcome of VITALITY-HFpEFT Trials Studies. Analyses results and 95% confidence intervals for the Kansas City Cardiomyopathy Questionnaire (KCCQ) physical limitation score (PLS) and the 6-minute walk test (6MWT) distance in the patients with LVEF≥45%. LVEF, left ventricular ejection fraction.

**Figure 3:**
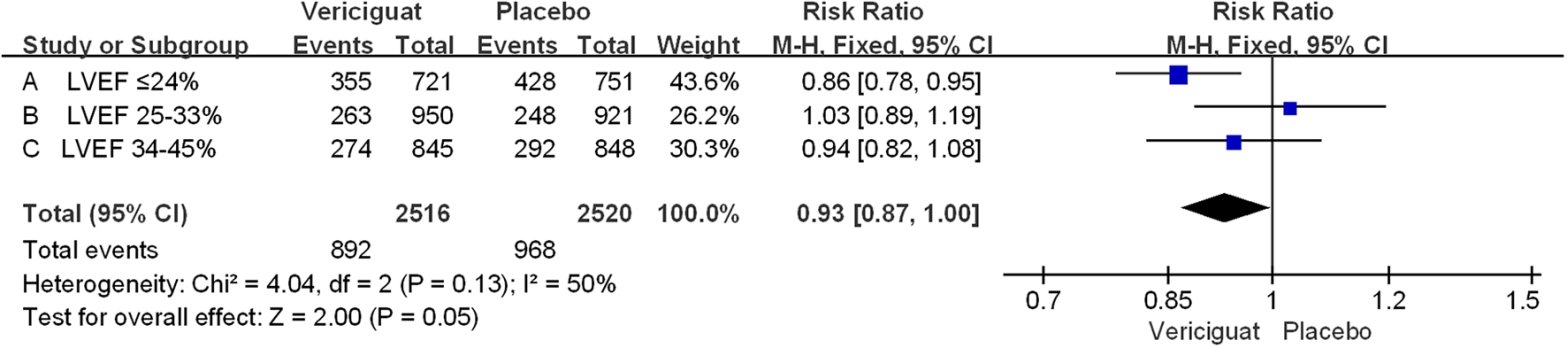
Primary outcome of VICTORIA Trials Studies. The Risk ratios and 95% confidence for the primary outcome (cardiovascular death or hospitalization for heart failure) of patients with different stages of left ventricular ejection fraction. LVEF, left ventricular ejection fraction.

In The VITALITY-HFpEF trial (LVEF≥45%) Studies, the Kansas City Cardiomyopathy Questionnaire (KCCQ) physical limitation score (PLS) was chosen as the experimental endpoint. The change in KCCQ PLS from baseline to week 24 was defined as the primary endpoint of this study (20). Because it is a direct measure of the hypothesized treatment effect, representing an improvement in functioning in activities of daily living limited by heart failure symptoms. This endpoint provides a valid and appropriate measure of the limitations on activities of daily living imposed by HF symptoms such as dyspnea and fatigue, which are a significant burden for patients with HFpEF (21). In our meta-analysis, we aimed to primary outcomes in the 6-minute walk test (6MWT) distance and the Kansas City Cardiomyopathy Questionnaire (KCCQ) physical limitation score (PLS) in the VITALITY-HFpEF trial (LVEF≥45%) in **Figure 2**. For the KCCQ PLS analysis, a total of 225 patients in the placebo group and 422 patients in the treatment group were analyzed. In patients with LVEF≥45%, the KCCQ PLS favored the experimental vericiguat group compared with the placebo group but was not statistically significant (mean difference 1.50; 95% CI -2.72 to 5.72). For the 6MWT distance analysis, the 6MWT distance favored the experimental vericiguat group compared to the placebo group but was not statistically significant (mean difference 3.50; 95% CI -15.36 to 22.36). Combined analyses showed no significant difference between vericiguat treatment and placebo in terms of KCCQ PLS and 6MWT analyses (mean difference 1.60; 95% CI -2.52 to 5.71). In the VICTORIA trial (LVEF<45%), the primary outcome was cardiovascular disease or first heart failure hospitalization as a composite outcome (22). The secondary outcomes included the components of the primary outcome, first HF hospitalizations, a composite of all-cause death or first HF hospitalization, and all-cause death in **Figure 3**. For patients with LVEF ≤ 24%, the incidence of the primary outcome was low in the vericiguat group and statistically significant (RR 0.86; 95% CI 0.78 to 0.95); for patients with LVEF in the range of 25-33%, the incidence of the primary outcome was low in the control group compared with the vericiguat group but not statistically significant (RR 1.03; 95% CI 0.89 to1.19); for patients with LVEF in the 34-45% range, the primary outcome incidence was low in the vericiguat group, but not statistically significant (RR 0.94; 95% CI 0.82to1.08). Combined analyses showed a lower incidence of the primary outcome in the experimental vericiguat group than the control group among all experimenters with LVEF<45%, significantly more in patients with LVEF<45% (RR 0.93; 95% CI 0.87 to 1.00).

**Figure 4:**
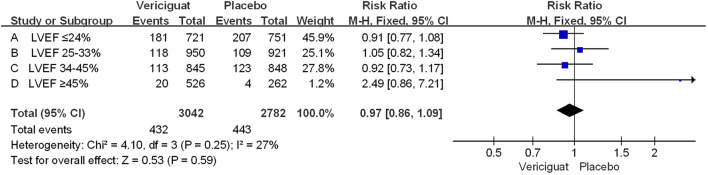
The CV death of VICTORIA and VITALITY-HFpEF Trials Studies. The Risk ratios and 95% confidence for the CV death (cardiovascular death) of patients with different stages of left ventricular ejection fraction. LVEF, left ventricular ejection fraction.

In addition, we performed a statistical analysis of the occurrence of cardiovascular death (CV death) in the two studies in **Figure 4**. We could see that among all experimental patients with LVEF<45%, the incidence of CV death was overall lower in the vericiguat group compared with the control group, but there was no statistical difference. Whereas, among patients with LVEF≥45%, the CV death incidence was instead higher in the vericiguat group, but also not statistically different. Overall, across the LVEF range, the incidence of CV death was lower in the vericiguat group than the placebo group, but there was no significant difference (RR 0.97; 95% CI 0.86to1.09). In summary, although the VITALITY-HFpEF and VICTORIA Trials Studies have different primary outcome settings, both are reasonable indicators of judgment (8, 17, 23); on this basis, combining the two clinical studies, we can conclude that no deterioration occurs in patients taking vericiguat across the entire LVEF range, but instead there is a significant benefit in improving clinical outcomes in patients with reduced LVEF (LVEF<45%), significantly more so in patients with LVEF ≤ 24%.

### Effect of vericiguat on serious adverse events according to ejection fraction

**Figure 5:**
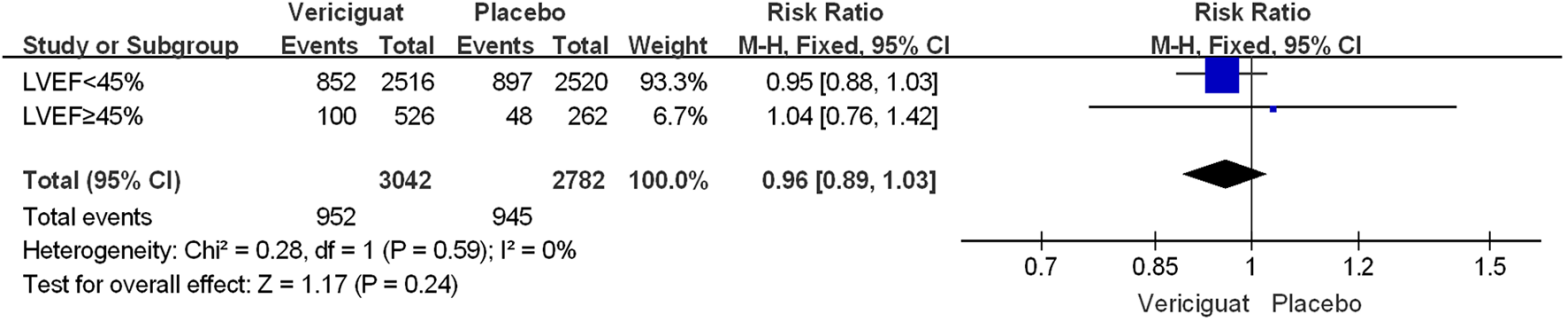
The serious adverse events of VICTORIA and VITALITY-HFpEF Trials Studies. The Risk ratios and 95% confidence for The Serious Adverse Events of patients with LVEF<45% and LVEF≥45%. LVEF, left ventricular ejection fraction.

**Figure 6:**
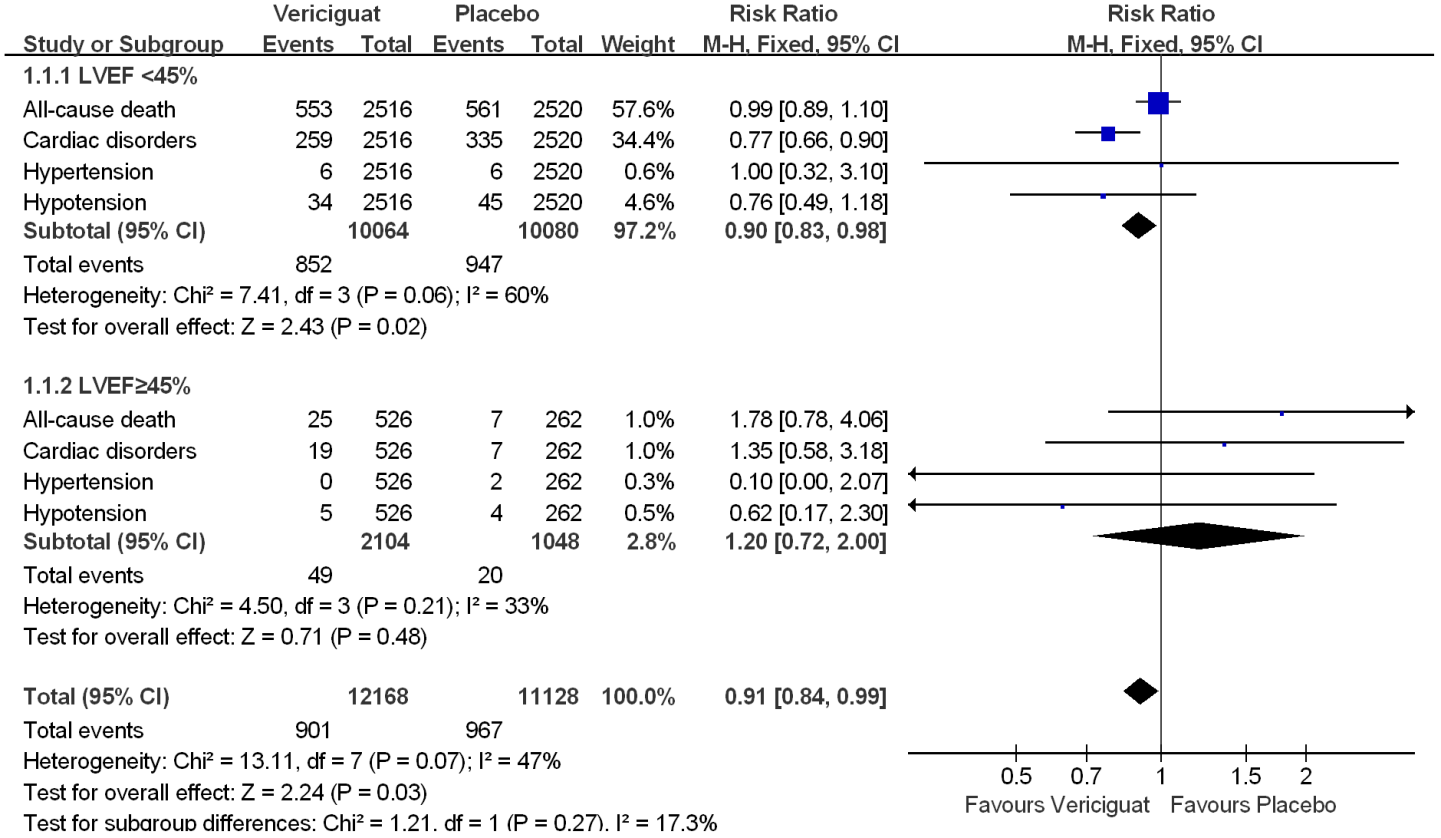
The partial subgroups analysis of serious adverse events in VICTORIA and VITALITY-HFpEF Trials Studies. The Risk ratios and 95% confidence for the All-cause death, Cardiac disorders, Hypotension, and Hypertension Events of patients with LVEF<45% and LVEF≥45%. LVEF, left ventricular ejection fraction.

To compare the effects of vericiguat treatment in patients within different LVEF, we also statistically analyzed the occurrence of serious adverse events in patients in both studies. Firstly, in the total adverse events in **Figure 5**, we found that in patients with LVEF<45%, the incidence of Serious Adverse Events was significantly lower in the vericiguat group compared with the control group, but not statistically different (RR 0.95; 95% CI 0.88 to 1.03); and in patients with LVEF≥ 45% in control and vericiguat groups had almost no difference in the incidence of Serious Adverse Events (RR 1.04; 95% CI 0.76 to 1.42); the overall analysis found that the low incidence of Serious Adverse Events was more skewed toward the experimental vericiguat group, but there was still no significant heterogeneity (RR 0.96; 95% CI 0.89 to 1.03). In summary, although it cannot be shown that the experimental vericiguat group reduces the incidence of Serious Adverse Events, it can be confirmed that the vericiguat group does not increase the incidence of serious adverse events. In addition, we performed the partial subgroup analysis of serious adverse events in VICTORIA and VITALITY-HFpEF trial studies in **Figure 6**. To our surprise, the overall incidence of the four serious adverse events, All-cause death, Cardiac disorders, Hypotension, and Hypertension, was significantly lower in the vericiguat group than the control group with LVEF<45% and was significantly different (RR 0.90; 95% CI 0.83 to 0.98); in particular, the incidence of Cardiac disorders was significantly lower in patients with LVEF<45% compared with the placebo group and was significantly different (RR 0.77; 95% CI 0.66 to 0.90). In contrast, in patients with LVEF≥45%, the total incidence of the four serious adverse reactions did not differ significantly between the experimental vericiguat group and the control group (RR 1.2; 95% CI 0.72 to 2.0). The final overall analysis showed that these four serious adverse reactions were still significantly lower in the vericiguat group than the control group over the entire LVEF range (RR 0.91; 95% CI 0.84 to 0.99).

In conclusion, although two clinical studies have shown that treatment with vericiguat does not significantly reduce the overall incidence of adverse events in patients with HF, treatment with vericiguat minimizes the incidence of all-cause death, Cardiac disorders, Hypotension, and Hypertension in patients with LVEF<45%, and in particular has a significant reduction in the incidence of Cardiac disorders.

## Discussion

Several studies have confirmed the beneficial effects of Vericiguat in patients with HFrEF, including the reduction of the incidence of heart failure progression and the prolongation of survival (15, 17, 18, 24). However, there are few comparative studies on whether Vericiguat has any benefit in patients with a full range of LVEF. This meta-analysis is the first to combine two extensive clinical studies, VICTORIA (LVEF<45%) and VITALITY-HFpEF (LVEF≥45%), to analyze the therapeutic effect of Vericiguat in patients with internal failure in the full range of LVEF. First, our comparative analysis found no difference in the primary outcome between the experimental Vericiguat group and the placebo group compared with the placebo group in VITALITY-HFpEF (LVEF≥45%), whereas the primary outcome (CV death or hospitalization for heart failure) in VICTORIA (LVEF<45%) was significantly improved by the administration of Vericiguat, which did not improve CV death alone; In the full LVEF range, vericiguat had no significant effect on the incidence of CV death or total serious adverse events compared with the placebo group. Surprisingly, the partial subgroups analysis of serious adverse events found that treatment with vericiguat reduced the incidence of four serious adverse events, namely All-cause death, Cardiac disorders, Hypotension, and Hypertension, in patients with LVEF<45%, especially in the incidence of Cardiac disorders. Taken together, Vericiguat showed a significant benefit in the treatment of HF patients with LVEF<45%, with the most significant benefit especially in patients with LVEF<24%, and a less pronounced therapeutic effect in HF patients with LVEF≥45%, but no adverse effects were found.

Recent studies have shown that different drugs are recommended for patients at different LVEF stages and can achieve different therapeutic outcomes (25); due to the predominant number of HFrEF patients, most drug studies are now focused on HFrEF, while studies of drugs for HFpEF patients are missing (26). However, the LVEF of patients with HF changes over time during treatment, and patients with HFpEF and HFrEF transform into each other. In the absence of real-time detection of patients’ LVEF, it is essential that medications that are effective in patients with HFrEF, even if they are not effective in patients with HFpEF, do not have a harmful outcome impact on patients with HFpEF (27, 28). The NO-sGC-cGMP axis is essential for the regulation of the cardiovascular system and the improvement of cardiac function in heart failure (29–32); earlier studies have demonstrated that the exogenous NO drug nitroglycerin can ameliorate angina, but it has the disadvantage of high first-pass metabolism, a low half-life and no specific effect (33); Inhibition of phosphodiesterase (PDE) activity downstream of cGMP has emerged as a new strategy for the use of sildenafil treatment (32). However, this pathway only inhibits degradation at the end and has limitations in therapeutic efficacy. Therefore, the question of whether the only known NO receptor, sGC, can be used as a target for developing corresponding drugs has attracted attention (34, 35). sGC is partially activated when NO in the body binds to heme (36). Stimulation of sGC activation has increased cGMP content, thereby improving pathological cardiac remodeling (37).

The sGC stimulator, the novel drug vericiguat, affects the NO-sGC-cGMP pathway to improve cardiac function in patients with heart failure (38). This new mechanism of action provides new options for patients and reduces the risk of deterioration (39, 40). At the same time, vericiguat avoids many problems, such as drug dose-dependent tolerance, progressive decline in effectiveness, and off-target effects due to lack of specificity (41, 42). However, there are fewer comparative studies of vericiguat in the full LVEF patient range, and previous studies have confirmed that vericiguat improves LVEF reduction in patients with CV death and heart failure hospitalization (43). Our study found that vericiguat improved the composite primary outcome (cardiovascular death or hospitalization for heart failure) in patients with reduced LVEF but was not found to reduce CV death in patients with reduced LVEF when analyzed separately. Overall, our comprehensive comparative analysis of two extensive clinical studies, VICTORIA (LVEF<45%) and VITALITY-HFpEF (LVEF≥45%), revealed the therapeutic impact of vericiguat in patients across the full LVEF range, further elucidating the therapeutic efficacy of vericiguat in patients with reduced LVEF, particularly significant in patients with LVEF<24%. In addition, vericiguat does not have a significant therapeutic effect in HF patients with LVEF≥45%. Still, it also does not have the unfavorable impact of increasing the number of serious adverse events, etc.

The results of this study confirm that vericiguat seems more efficacy in HFrEF patients (LVEF <45%) compared to HF with LVEF > 45%, which may be related to the mechanism of action of vericiguat in the heart. Vericiguat exerts cardioprotective effects by stimulating sGC. The expression of sGC is the highest in cardiomyocytes. The cGMP produced by sGC causes ventricular relaxation, reduced contractility, and has anti-hypertrophic and anti-fibrotic effects (44). The myocardial effects of the NO-sGC-cGMP pathway may be due to the regulation of titin, a major determinant of myocardial stiffness. The NO-sGC-cGMP pathway is impaired in HFrEF, which is characterized by neurohormonal activation and systemic vasoconstriction that overwhelms NO-sGC-cGMP-mediated vasodilation (45). HFrEF is also associated with endothelial dysfunction caused by oxidative stress, which leads to reduced endothelial NO synthase activity and absolute NO deficiency. The function of the NO-sGC-cGMP pathway depends on the body’s redox status. Increased oxidative stress in HFrEF disrupts signaling cascades through varying degrees of NO, sGC, and cGMP inactivation, with downstream effects including increased vascular tone, stiffness, afterload, and left ventricular pressure, impaired coronary microcirculation, and myocardial infarction. Cells are susceptible to ischemic damage (45). In summary, the effective effect of vericiguat on patients with HFrEF is based on stimulating sGC to improve ventricular systolic and diastolic ability, which also corresponds to its effectiveness in patients with decreased LVEF.

The European Society of Cardiology’s recently released HF treatment guidelines emphasize four “key disease-modifying drugs”, mainly including beta-blockers, renin-angiotensin-aldosterone system inhibitors, and mineralocorticoid receptor antagonists and sodium-glucose cotransporter 2 inhibitors (46). However, residual risks still exist after using these four drugs. In view of the complexity of pathological changes in the development of heart failure, it is particularly important to explore new complementary therapeutic drugs that are different from the above four drugs and are effective for HF, so that we can hope to achieve the goal. The goal is to significantly improve and reverse HF. Vericiguat is an sGC stimulator that works by increasing the production of cGMP, a molecule that promotes vasodilation and reduces oxidative stress and inflammation. Vericiguat has a different mechanism of action from the four “key disease-modifying drugs” and is expected to become the fifth key drug in the treatment of HF (41, 47, 48).

In conclusion, based on indirect comparisons, vericiguat significantly improved the primary outcome (CV death or first heart failure hospitalization) compared with placebo in HF patients with LVEF<45%. The risk of the four serious adverse events of All-cause death, Cardiac disorders, Hypotension, and Hypertension was lower, especially Cardiac disorders. Vericiguat did not significantly improve the HF patients with LVEF≥45%, and there was no significant difference in the occurrence of serious adverse events and CV death in patients with HF compared with placebo.

### Limitation

The primary outcome measures for VICTORIA and VITALITY-HFpEF were not the same, but both are recognized as valid outcome indicators; in addition, there was a significant difference in the number of people in the two studies, but both were also consistent with the statistical sample size of dogs in extensive clinical studies.

## Author contributions

CC: Data curation, Investigation, Writing – original draft, Writing – review & editing. CL: Funding acquisition, Resources, Supervision, Writing – review & editing. JL: Methodology, Writing – review & editing.
